# Return of the cadaver: Key role of anatomic dissection for plastic surgery resident training

**DOI:** 10.1097/MD.0000000000007528

**Published:** 2017-07-21

**Authors:** Swenn Maxence Krähenbühl, Paul Čvančara, Thomas Stieglitz, Raphaël Bonvin, Murielle Michetti, Marjorie Flahaut, Sébastien Durand, Lina Deghayli, Lee Ann Applegate, Wassim Raffoul

**Affiliations:** a Plastic, Reconstructive and Hand Surgery Division, CHUV – Lausanne University Hospital, Lausanne, Switzerland; b Laboratory for Biomedical Microtechnology, University of Freiburg, Freiburg im Breisgau, Germany; c Education Unit, CHUV – Lausanne University Hospital, Lausanne, Switzerland.

**Keywords:** electrode implantable device, fresh cadaver dissection, nerve dissection, neuronal interface, plastic surgery, resident training

## Abstract

Successful Plastic Surgery Residency training is subjected to evolving society pressure of lower hourly work weeks imposed by external committees, labor laws, and increased public awareness of patient care quality. Although innovative measures for simulation training of surgery are appearing, there is also the realization that basic anatomy training should be re-enforced and cadaver dissection is of utmost importance for surgical techniques.

In the development of new technology for implantable neurostimulatory electrodes for the management of phantom limb pain in amputee patients, a design of a cadaveric model has been developed with detailed steps for innovative transfascicular insertion of electrodes. Overall design for electrode and cable implantation transcutaneous was established and an operating protocol devised.

Microsurgery of the nerves of the upper extremities for interfascicular electrode implantation is described for the first time. Design of electrode implantation in cadaver specimens was adapted with a trocar delivery of cables and electrodes transcutaneous and stabilization of the electrode by suturing along the nerve. In addition, the overall operating arena environment with specific positions of the multidisciplinary team necessary for implantable electrodes was elaborated to assure optimal operating conditions and procedures during the organization of a first-in-man implantation study.

Overall importance of plastic surgery training for new and highly technical procedures is of importance and particularly there is a real need to continue actual cadaveric training due to patient variability for nerve anatomic structures.

## 1 Introduction

Surgical training is within an era of continual emerging complexities and there is also the pressure of public awareness toward surgical competence. To be a successful surgeon in today's culture, technical proficiency is of utmost importance. However, there are associated qualities that should be acquired for the competent academic surgeon including overall medical knowledge, research activities, communication skills, personality, ability to work in a team, and commitment to professional and academic development. These elements associate together to assure the state-of-the-art level of knowledge of the surgeon in order to provide the best surgical potential and capacity to transmit and teach techniques.

However, all of these acquired qualities involve a high investment of time and cost. In 2003, the Accreditation Council for Graduate Medical Education (ACGME) in the USA restricted a resident's work week to 80 hours with at least 1 full day on leave per week.^[1–8]^ An additional 8 hours can be added for education and research purposes. The restrictions also wanted to assure a maximum of 30 hours of consecutive time at work. Further restrictions were added in 2011 for first-year residents to limit to a 16-hour work period with on-site supervision available, but more senior residents could have up to a 24-hour consecutive working assignment but with various other limitations to assure appropriate rest. One could imagine that decreasing time to overall practice would also decrease continuity of care, decreased education quality, and lower training possibilities. It has been well summarized by Luce^[4,5]^ that by reforming and focusing on the maximum time at work has shifted the emphasis from education to only a service function, whereas the overall goal should be extensive and proper training to allow for independent care of patients when the residency is completed.^[4,5]^ Following all the emphasis on hourly restriction, it was found several years after the time restriction imposition that there were no major differences in overall medical errors made by doctors. Over 142,000 people die every year in the US from medical errors.^[9]^ By centralizing changes on working hours alone by different oversite Committees would have led to oversight of other major issues such as lack of adequate supervision, nonadapted or no reliable computerized records, and most of all the training of efficient and appropriate communication of patient care between resident shifts or “hand-off” summaries for important patient parameters. Overall, the hourly restriction will relate to over 1000 less hours of training per year during residency in the USA.^[7,8]^

In Plastic Surgery, the time of training to acquire all surgical techniques and assure appropriate management of the various pathologies is extensive. In Switzerland, there has been implementation of the 2005 Swiss labor law that restricts working hours to only 50 a week for the medical doctors. Research and education are to be included in this 50 a week resulting in a 42-hour on-hands work week for many.^[10]^ Although a survey accomplished in 2013 in Swiss Hospitals revealed that almost 70% of Swiss MDs worked more than the 50 hours imposed level, more than 25% said they worked over 60 hours per week. This massive decrease of time for training can be associated with a large decrease in the total number of operations performed by plastic surgery residents and this decrease in operative volume exposure has been accompanied by diminished self-reported operative confidence and expert rating of the operative ability of recent graduates (clinical observations).^[11]^ Nowadays, to be formally prepared as a plastic surgeon, it has been observed that the number of years as a resident will have to increase to have full training. This will then have certain implications for overall training in the field of plastic surgery such as lower number of positions available, as current residents occupy the posts longer to enable full overall training throughout the full program required for both the European and Swiss board examination.

Because of deficiencies, innovative approaches with extensive computer simulation are now emerging to help train plastic surgeons even more.^[12]^ Other programs for hand surgery technique simulations but using imitation modeling instead of computers were developed for training in microsurgery and acquisition of necessary procedures to improve technical skills in hand trauma. The study evaluated the use of simulation and training for 4 tasks (Z-plasty, fixation of a metacarpal fracture, tendon repair, and end-to-end anastomosis) and was able to show that higher practice was indeed of significant benefit for surgical residents.^[13]^ These simple simulation models could easily be integrated into training and help increase technical practice. Surgical resident's competency for operating experience has been largely associated with adequate anatomy training and lack of such training can have an effect on their overall competence and associated confidence.^[14]^ Furthermore, in a survey conducted with Directors of US surgical fellowship programs, they reported that two-thirds of new residents could not attain more than 30 minutes of unsupervised operating room time and more than 20% were clearly not prepared for operating room procedures.^[14]^

The dissected body clearly has the potential to teach many lessons both to the undergraduate and the graduate medical doctors. Therefore, development of precise skills for dissection of cadaver models provides a valuable opportunity to exercise technique in a stress-free environment. However, detailed anatomical dissection is very time-consuming and revised undergraduate and graduate teaching have decreased hours in the use of cadavers by associating with other computer simulation. Unfortunately, some medical schools in Australia and the United Kingdom have even eliminated use of cadaver models altogether, even though studies have shown that it would be better to balance the use of digital technologies with cadaver dissection.^[12,15,16]^ In a study in Michigan State University, it was clearly shown that practical dissection of cadaver enhanced identifying body parts and also how they functioned when compared with groups of students who learned with multimedia and then were tested on cadaver models.^[12]^

Moreover, with the increasing number of medical students and development of alternative techniques, access to cadavers has become very difficult. For instance, there are some countries that have problems for cadaver acquisition due to religion, culture, or lack of information on organ donation.^[15,17,18]^ Therefore, anatomists worldwide at the International Federation of Associations of Anatomists (IFEA) meeting in 2014 in Beijing proposed to create an International Network to help countries with difficulty in setting up donation programs.^[17]^ They have gone 1 step further by proposing principles for good practice of body donation and to have exchanges between countries for optimization of full body or body parts to be used in medical teaching.^[19]^ The anatomists, pathologists, surgeons, and ethicists can all work together to utilize the dissected body to its fullest extent.

Anatomical dissection sessions on fresh cadavers with our staff surgeon assisted with a resident were thus organized for the implantation and a dissection course for plastic surgery residents was thus elaborated. A training model of anatomic dissection on fresh cadavers to enhance surgical technique and operative confidence was elaborated for inter-fascicular nerve dissection and electrode implantation. These proposed techniques are new and thus need to be implemented first in specific training. In the framework of a European Program for research and technological development (FP7-EPIONE) project, the feasibility to implant microelectrodes into the upper limb nerves-median and ulnar was to be developed and assessed. The electrodes were newly developed for this purpose. These electrodes are to stimulate the nerves when attached to an electronic stimulator with the overall goal aiming to assess the natural feedback for phantom limb pain modulation and therapy in amputee patients (http://project-epione.eu).

This experience that was motivated through a research project portrayed the importance of cadaver dissection and has resulted in a clear motivation for “the return of the cadaver,” even though this type of training is more time-consuming and costly.

## 2 Methods

### 2.1 Cadavers

Cadaver specimens (4 upper limbs) were obtained from the Anatomy Department of the University Hospital of Lausanne under authorization of organ donations. These were obtained under the legal and ethical framework governing donation with a harmonization of the use of cadavers for anatomical sciences and under specific protocols.^[17]^ The overall set-up, material, and techniques were reviewed and developed with the Chief of Plastic Surgery and first-year Residents.

### 2.2 Electrodes

**Figure 1:**
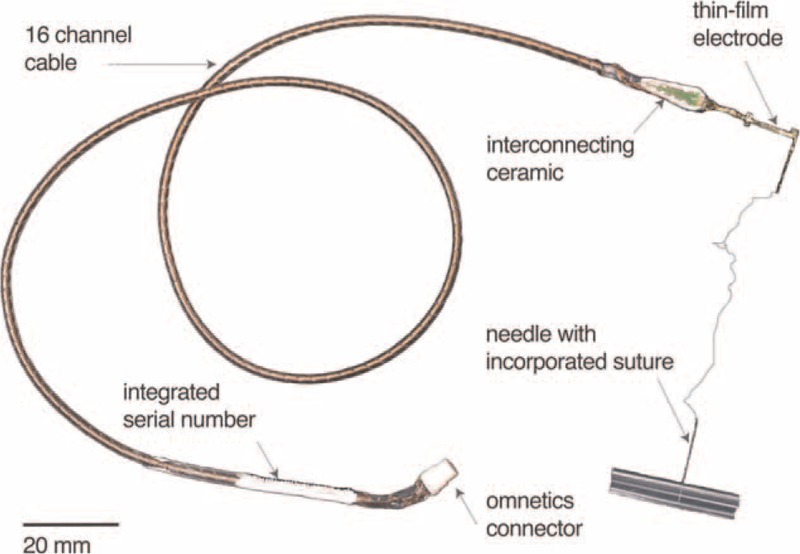
TIME-4H electrode and components. Assembled TIME-4H electrode system with helical cable, polyimide electrode with needle, and Omnetics connector.

The transverse intrafascicular multichannel electrode (TIME-4H, latest version, developed by Laboratory for Biomedical Microtechnology, IMTEK, University of Freiburg, Germany) is based on a polyimide thin-film substrate with incorporated metal tracks and contact sites (Fig. 1). The thin-film part is micro fabricated with standard photolithographic processes in a class 100 cleanroom environment. To ensure a sufficient charge injection, the 14 active contact sites and two ground electrode sites are coated with highly porous sputtered iridium oxide film (SIROF). The thin-film part has an overall thickness of about 11 μm and is attached via the microflex interconnection technique to a screen printed ceramic. This ceramic is soldered to a 40 cm long cable (Fig. 1), which is made of 16 helically wound MP35N (NiCo base alloy) wires in a polyesterimide (PEI) jacket, covered with a medical grade silicone tubing and filled with silicone rubber. The cable is terminated with a 16-pole Omnetics Nano Circular connector (NCP-16-DD, Omnetics, Minneapolis, US), having a diameter of 4.6 mm, which is used to contact the electrodes’ active sites electrically. Within the thin-film electrode, a guiding needle with a loop of suture (EH7900G, Prolene, Ethicon, Johnson & Johnson, NJ) is incorporated having a diameter of 125 μm and a suture length of 15 cm. The risk assessement dossiers has been evaluated with the STIMEP stimulator (Axonic, Sophia Antipolis, France) only.

## 3 Results

### 3.1 Cadaver positioning for upper extremity nerves

The arm was positioned at 90° and rotated externally with the elbow slightly flexed.

The skin incision followed the internal bicipital groove for a total length of about 15 cm. The brachial fascia was incised along the medial border of the biceps muscle, remaining anterior to the medial intermuscular septum.

**Figure 2:**
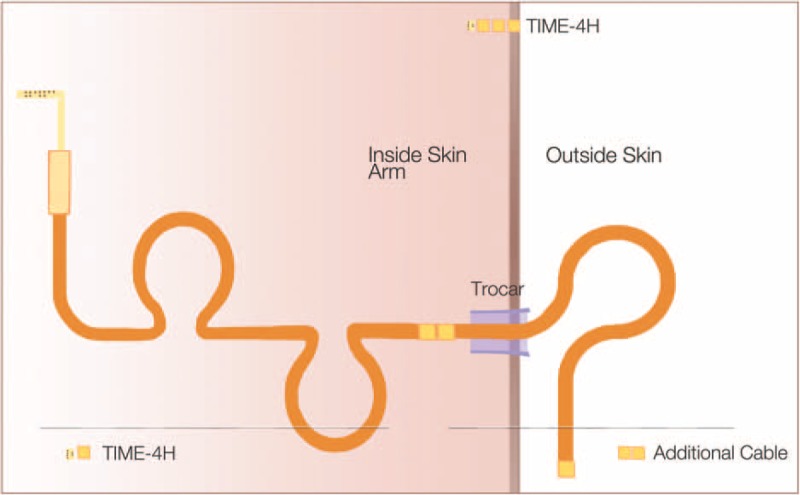
Diagram of TIME-4H positioning. TIME-4H electrode passed through the skin using a trocar (10 mm) to protect material transfer. Double loops of excess cable are implanted internally to allow for patient movement and less tension on transplanted electrode.

The plastic surgeon then moved the biceps muscle laterally and the triceps muscle medially in order to expose the neurovascular bundle of the arm for identifying the anatomical structures, including the median nerve, ulnar nerve, brachial artery, and medial cutaneous nerve of the forearm (Fig. 2).

### 3.2 Median and ulnar nerve dissection

The nerves of interest were the median and ulnar nerves that were then dissected from the surrounding tissue for the entire length of the wound opening. Four tissue spaces were needed to be prepared above the muscular plan for the accommodation of TIME electrode cables. There were 2 lateral spaces prepared for the electrodes implanted within the median nerve and 2 medial spaces prepared for the electrodes implanted within the ulnar nerve (Fig. 2).

### 3.3 TIME-4H implantation

**Figure 3:**
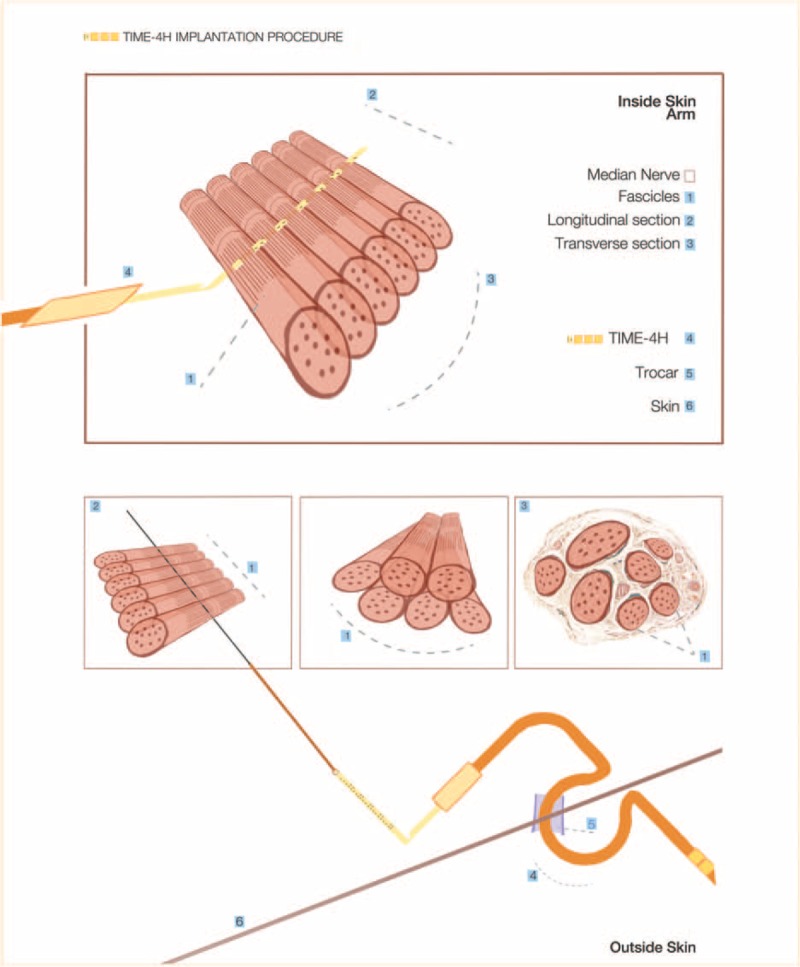
Interfascicle nerve electrode implantation. Illustration of specific fascicle separation and insertion of electrode through each individual nerve fascicles using a defined needle with incorporated suture.

**Figure 4:**
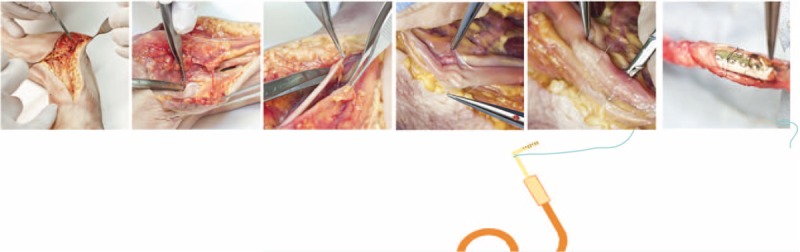
Cadaver model development. Incision of skin and associated muscles followed by dissection of individual nerves was first prepared. Nerve sheath was opened and individual fascicles were penetrated one-by-one with the positioning needle to align the TIME-4H and fix the ceramic adaptor to the nerve.

Once the TIME electrode cables were placed within a trocar (Fig. 3) that prepared transversal positioning, the rectangular connector of the electrode (interconnect block) could be placed along the nerve. Each nerve was dissected along its length for approximately 2 cm and opened in a book-like fashion to display all of the nerve fascicles (Fig. 4). With the aid of the intraoperative microscope (Carl Zeiss Meditec AG, Jena, Germany), the surgeon placed the entry point of the TIME within the first nerve fascicle and aligned each of 4 to 6 remaining fascicles to insert the electrode consecutively in each of the nerve fascicles (Figs. 2 and 4). A guiding needle and filament was used to place the active sites inside the nerve. The guiding needle that was used was an Ethicon needle (125 μm diameter, 26° tip angle) (Ethicon, Johnson & Johnson, NJ) with a pre-attached filament and was pulled by the TIME through the wire until the active site of the electrode would be placed in the correct position inside the nerve fascicles. These maneuvers were carried out with great caution to avoid the wire breaking at the connection point with the electrode. Because of the surgical microscope, the surgeon could check the correct positioning of the active sites of the TIME inside the fascicles.

An 8-0 suture was used to tie the electrode to the epineurium through the 3 fixation holes.

### 3.4 Anchor of electrodes and cables

This connector was finally anchored to the surrounding nerve sheath with nonabsorbable 4-0 silk threads, so as to prevent that any twisting forces applied to the electrode cable would reposition the TIME.

The ceramic interconnect of the electrode was directly anchored by suturing to the nerve as shown in Fig. 2, as this would not induce any compression and it would avoid any problem for the interface of the electrode embedded into the connective tissue from detaching from the nerve.

The flexible electrode cable for external connection with the stimulator was housed with 2 loops within the previously prepared subcutaneous tissue spaces in order to reduce the tensile forces on the electrode. The loops were then anchored to the muscle and facial tissue with 4-0 silk wire suture and the wires were externalized percutaneously to enable the electrodes to subsequently be connected with the stimulator.

The procedure could be repeated for the other 3 electrodes (a total of 2 on the median nerve and 2 on the ulnar nerve) taking care to avoid intersections between the electrodes and the output cables. Each TIME has an external marking with different colors to ensure easy identification.

At this point, the 3 anchorage holes of the TIME could be sutured to the epineurium through nonresorbing 8-0 wires (Fig. 2). The full procedure and training requires 3 to 4 hours for both macro- and microsurgical processes.

### 3.5 Operating arena descriptive and positioning

**Figure 5:**
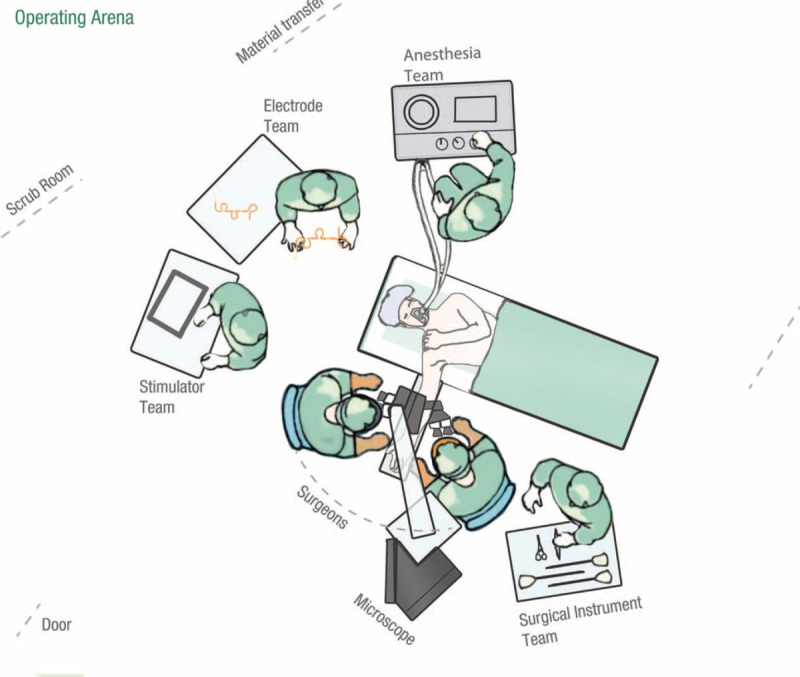
Optimization of operating room. Surgical team positioned on each side of the arm to have nerve dissection and electrode implantation. The Stereomicroscope is positioned at the base of the operated arm. Surgical instrument team is located behind the Senior Surgeon. Anesthesia team is on the opposing side of the surgical team. At the head of the patient and operating table are the 2 teams for technical support for both the electrodes and stimulator.

Optimal operating arena positioning for the multidisciplinary team was prepared for a standard operating procedure for dissection of nerves and inter-fascicular nerve implantation. The surgical team is positioned on each side of the arm to have nerve dissection and electrode implantation (Fig. 5). The Stereomicroscope is positioned at the base of the operated arm. Surgical instrument team is placed behind the Senior Surgeon and Resident and the anesthesia team is on the opposing side of the surgical team. At the head of the patient and operating table are the 2 teams for technical support for both the electrodes and stimulator (Fig. 5).

## 4 Discussion

Developing detailed protocols using cadaver models will help to eliminate uncertainty and can then be accomplished under conditions that are less stressful than when the new technique is used on patients in the operating room for the first time. By successfully preparing all steps of new operating protocols, it is possible to optimize knowledge for the entire team. Especially for Plastic Surgery, anatomical dissection particularly of peripheral nerves and free flap surgery allows no margin for error. These programs are very important for Plastic Surgery training where repeated dissection and preparation of detailed protocols can be accomplished for new techniques.^[20,21]^ Indeed, preliminary observations of a first patient who has been operated with this new technique of inter-fascicle nerve implantation a year ago have been found to be safe with no serious side effects (personal communication EPIONE consortium, Swiss Ethics approval: #PB_2016_02263). Overall, evolving experience for improving surgical techniques and educational activities should be described routinely and to provide key opinions for future studies and training. This has been reported recently by the group of Fong et al^[22]^ in describing operating room efficiency. They have emphasized the importance of the surgeon for procedural efficiency with their key role for team communication and standardizing tasks that seem to be primordial in surgical training.^[22]^ The value of anatomy dissection courses using human fresh cadavers in imparting anatomy knowledge has been clearly established. The return of basic training techniques for surgeons such as cadaver dissection is shown to be of high value not only for research and development but also for the valuable hands-on experience. Nowhere is this more important than for micro-dissection techniques such as for peripheral nerves.^[23]^ Cadaver dissections are particularly important for nerves in the upper limbs, as these structures can vary from person to person.^[24]^ The practice of defined techniques is especially important for new procedures.

In the framework of the FP7-EPIONE project, the feasibility to implant microelectrodes into the upper limb nerves-median and ulnar was to be developed and assessed. For this purpose, micro-dissection of the nerve was established as an alternative technique for the electrode implantation instead of the technique of passing the electrode through the entire nerve where only few fascicles would be included. Positioning of all of the electrodes and cables was accomplished with respect to the anatomical location to avoid first, damage during implantation, and second to avoid movement problems postimplantation. In addition, the positioning of the multidisciplinary team within the operating room was defined to allow a better flow. Microsurgical techniques are becoming more important for nerves, as there are more and more implantable devices being proposed, and therefore, the development of such devices should be done hand-in-hand with the surgeons. Innovative technologies emphasize the importance for efficient collaboration between engineers and surgeons to succeed in new protocol implementation.

## 5 Conclusion

We have emphasized the use of a cadaveric model for developing new and innovative methodology to implant very small electrodes more efficiently within a nerve by dissecting fascicles and implanting the electrodes into each one individually. Inter-fascicular implantation for electrostimulation could have better results than having them implanted randomly through different numbers of fascicles.^[24]^ The microsurgery technique could be practiced on cadaver models and detailed operating protocols could be established before beginning with a first patient. As a new microsurgical technique was to be introduced, cadaveric models were once again positioned with a high value and thus we enter into a new scenario of “the return of the cadaver” to emphasize perhaps their importance for routine teaching again.
